# Phosphorylation event changes the RNA binding mode of EZH2 disordered segment

**DOI:** 10.1002/pro.70391

**Published:** 2025-12-23

**Authors:** Beáta Szabó, András Micsonai, József Kardos, Ágnes Tantos

**Affiliations:** ^1^ Institute of Molecular Life Sciences, Center of Excellence of the Hungarian Academy of Sciences HUN‐REN Research Centre for Natural Sciences Budapest Hungary; ^2^ ELTE‐Functional Nucleic Acid Motifs Research Group, Department of Biochemistry, Institute of Biology ELTE Eötvös Loránd University Budapest Hungary; ^3^ Department of Biochemistry, Institute of Biology ELTE Eötvös Loránd University Budapest Hungary; ^4^ ELTE NAP Neuroimmunology Research Group, Department of Biochemistry, Institute of Biology ELTE Eötvös Loránd University Budapest Hungary

**Keywords:** circular dichroism, EZH2, HOTAIR, intrinsically disordered protein, lncRNA, microscale thermophoresis, PRC2, protein–RNA interaction

## Abstract

Polycomb repressive complex 2 (PRC2), which exhibits an important gene silencing role in development and differentiation, has been shown to interact with several long non‐coding RNAs (lncRNAs) that influence its function and determine its localization. One of the most prominent and researched lncRNA partners of PRC2 is HOTAIR, which is shown to direct the proper localization of the complex within the chromatin. While many important details of the recognition of RNAs by different complex subunits have been revealed, the exact molecular mechanisms remain elusive. EZH2, the methyltransferase subunit of PRC2, is one of the proteins that are responsible for RNA binding, containing multiple RNA binding surfaces. One of the binding interfaces lies in a disordered loop region of EZH2, where a phosphorylation event is known to increase RNA binding in cells. To reveal the molecular details of the effect of phosphorylation of the disordered loop of EZH2, we expressed the region and tested its interaction with several RNA constructs, including different segments of HOTAIR. We found that the EZH2 loop exhibits varying affinities towards different RNA species, with a limited sequence specificity. Phosphorylation did not have a significant effect on binding strengths, but it altered the structural background of the interaction. While the protein itself remained disordered in the bound form, the phosphomimetic mutant version of the EZH2 loop was capable of opening (unfolding) the double‐stranded RNA regions upon interaction. Our findings offer an explanation of the molecular mechanism of the RNA recognition by a disordered segment in EZH2 and clarify the assumed regulatory role of the phosphorylation event.

## 1 INTRODUCTION

EZH2 (Enhancer of zeste homolog 2) is the histone methyltransferase component of the Polycomb Repressive Complex 2 (PRC2), which is responsible for the trimethylation of the K27 residue of histone H3 (H3K27). This histone modification induces silencing of the related gene, and consequently, the activity of PRC2 is necessary for proper development and differentiation (Cao et al. 2002; Margueron and Reinberg 2011). Abnormal activity of EZH2 is often observed in different malignant processes, such as breast, lung, and pancreatic cancers (Kleer et al. 2003; Laugesen et al. 2016), and is related to disease progression (Kleer et al. 2003; Matsukawa et al. 2006). Due to its important roles in gene expression regulation and different diseases, the molecular mechanisms of the activity and targeting of PRC2 have been excessively studied. The search for specific factors that determine PRC2 localization to its target regions within the chromatin led to the recognition of the involvement of long non‐coding RNAs (lncRNAs) in this process. After the initial discovery that the lncRNA HOTAIR can interact with PRC2 and direct its targeting (Tsai et al. 2010), several additional details of the PRC2‐RNA interactions have been described. While other PRC2 complex components are also capable of RNA binding, EZH2 appears to have the highest affinity towards RNA in vivo (Betancur and Tomari 2015; Cifuentes‐Rojas et al. 2014). Although RNA binding by PRC2 appears to be rather promiscuous (Davidovich et al. 2013), specific RNAs have more prominent roles than others, raising questions about the details of RNA recognition (Davidovich et al. 2015).

HOTAIR is one of the most important lncRNA partners of PRC2 (Cerase and Tartaglia 2020), which is involved in the targeting of the complex (Tsai et al. 2010).

Numerous pieces of evidence support the in vitro binding of EZH2 to HOTAIR and it has been shown that the 5′ domain of HOTAIR is recognized by EZH2 (Kaneko et al. 2010). EZH2 appears to have multiple binding surfaces for RNAs, some of which specifically recognize G‐quadruplex structures (Long et al. 2017).

Because of the prominent roles of RNA binding in the regulation of PRC2 functions, the molecular features that govern the interactions on both sides have attracted significant scientific interest. Although EZH2 is devoid of canonical RNA recognition motifs, it harbors several, structurally diverse segments that can bind RNA in vitro, including regions between residues 32–42, 210–224, 488–510, 512–531, and 645–665 (Long et al. 2017). An additional, disordered loop, between residues 340–435, also serves as an RNA interaction surface (Kaneko et al. 2010; Szabó et al. 2022). Although all of these distributed patches exhibit different affinities toward RNAs and efficient binding is most probably achieved through their concerted action in the context of the complete PRC2 complex, their roles and relevance are broadly accepted. However, EZH2 recognition on the RNA part is a much more ambiguous and debated subject. Several different RNA structures have been proposed to be recognized by EZH2, including two‐hairpin motifs (Davidovich et al. 2015) and G‐quadruplexes (Long et al. 2017), but neither of these could be concluded to unequivocally describe specific RNA recognition by EZH2.

The relevance of this question is underlined by the recent results suggesting that RNA structure plays an even more important role in the activity of PRC2 than previously thought. It was proposed that single‐stranded RNA binding by EZH2 needs to be exchanged to double‐stranded, lower‐affinity interactions, promoting PRC2 interaction with chromatin (Balas et al. 2021). The conformation shift that allows this, happens through the interaction of HOTAIR and hnRNP B1, when the low‐complexity domain of the protein unwinds a helix region in HOTAIR (Kumar et al. 2023), initiating its interactions with other RNAs (Balas et al. 2021).

Moreover, RNA recognition may be modulated by posttranslational modification of the protein as well. Most prominently, a phosphorylation event at the T345 residue of EZH2 increases the affinity of EZH2 towards intracellular RNAs (Kaneko et al. 2010). Mechanistically, it was suggested that a small pool of EZH2 molecules phosphorylated in a cell cycle‐dependent manner may be responsible for the initial deposition of the H3K27me3 mark, which then gets propagated through the inherent H3K27me3 binding capacity of the EED subunit of PRC2 (Kaneko et al. 2010). While the importance of phosphorylation is also underlined by the independent observation that cyclin‐dependent kinases can regulate the activity of PRC2 and enhance its localization to its target loci (Chen et al. 2010), there is little information available on the molecular mechanism through which this modification influences EZH2–RNA interactions.

Our previous results showed that the EZH2 loop region binds HOTAIR without folding into a well‐defined structure, rendering the interaction fuzzy (Szabó et al. 2022), and the phosphorylation of the T345 residue did not induce large‐scale reorganization of the protein segment, offering little explanation for the effect observed in vivo (Kaneko et al. 2010). In order to further dissect the effect of phosphorylation on the RNA recognition of the EZH2 loop, we designed different RNA constructs and examined their interactions and their changes upon T345 phosphorylation. Based on previously published experimental results (Kaneko et al. 2010), we applied a phosphomimetic mutation, where the threonine residue was replaced with an aspartate (TD).

## 2 RESULTS

### 2.1 RNA binding of the EZH2 loop

**FIGURE 1:**
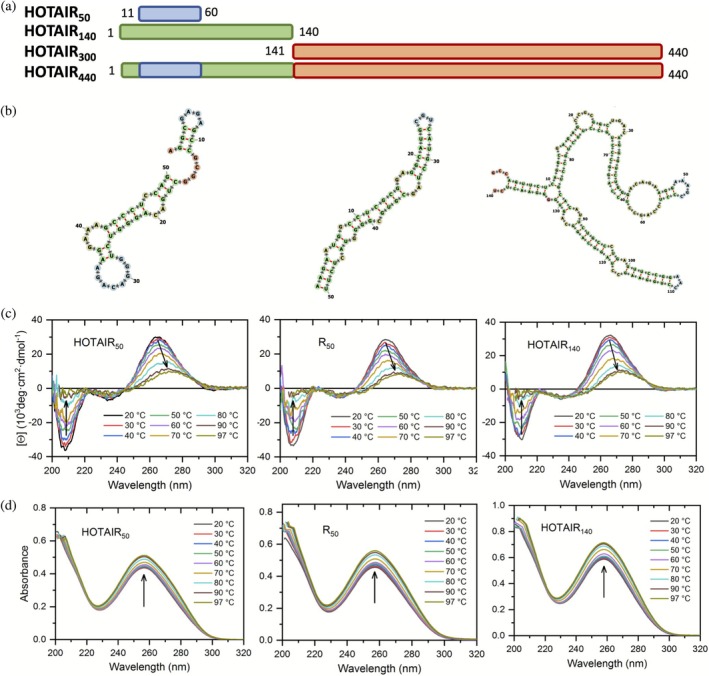
Structures of the HOTAIR constructs used in the work. (a) Schematic representation of the different sequences. (b) Predicted secondary structures of HOTAIR_50_, R50, and HOTAIR_140_. (c) CD spectra of the different RNAs and their temperature‐induced unfolding. (d) UV absorbance of the different RNAs as a function of temperature. Melting‐associated spectral shifts and amplitude changes are indicated by arrows.

To compare the RNA binding ability of the wild type and the phosphomimetic version of the EZH2 loop, we selected four different regions in HOTAIR (Figures 1a and S1a, Supporting Information). The shortest one, HOTAIR_50_, is a 50 nt‐long region between nucleotides 11 and 60. A 140 nt‐long sequence between nts 1 and 140 overlaps with HOTAIR_50_ and is adjacent to the sequence previously annotated as the EZH2‐binding segment of HOTAIR. The annotated EZH2‐binding region, HOTAIR_300_ is located between nts 141 and 440, and HOTAIR_440_ contains nucleotides between 1 and 440, encompassing all the shorter constructs. In order to test the sequence‐specific recognition capacity of the EZH2 loop, we used a 50 nt long, artificial RNA with randomized sequence as control (R50), designed to have no sequence similarity to the naturally occurring RNAs. Secondary structure predictors and the CD spectrum of the free HOTAIR_50_ indicate a hairpin‐like fold, with approximately 20 base pairings (Figure 1b,c). R50 has a highly similar structure to HOTAIR_50_, while the longer constructs adopt more complex structures (Figures 1b,c and S1b). We also chose to test a different lncRNA partner of EZH2, in order to clarify if the phosphorylation event increases its capability to differentiate between specific RNA partners. We chose Maternally Expressed 3 (MEG3) as the additional lncRNA, as it is a confirmed interactor of EZH2 (Li et al. 2022; Terashima et al. 2017; Wang et al. 2018) and has specific target genes, different from HOTAIR (Dunn‐Davies et al. 2024).

The CD spectra confirmed the presence of A‐form double‐stranded helix structure for all RNAs, characterized by a negative peak at 210 nm (Baranowski et al. 2015; Langkjaer et al. 2009) (Figures 1c and S1c).

The missing positive peak around 210 nm, the missing negative peak around 240 nm and the lack of sharp transition above 240 nm, characteristic of G‐quadruplexes (G4s) (Del Villar‐Guerra et al. 2018; Małgowska et al. 2012; Vorlíčková et al. 2012), and a strong broad positive peak at 265 nm. Stable G‐quadruplexes (G4s) contain two characteristic positive peaks at 210 and at 295 nm, and a negative peak at 245 nm, with a sharp transition above 240 nm (Małgowska et al. 2012). Since all of these are missing from the CD spectra of the RNA constructs studied here, our measurements suggest that these HOTAIR constructs do not contain stable G‐quadruplexes in significant portions, even though in silico predictions (Kikin et al. 2006) indicated the presence of weak G‐quadruplex structures in all constructs (Figure S1a). Increasing temperature resulted in the unfolding of the RNAs, accompanied by the loss of signal in the 210 nm peak and a reduction and shift of the 265 nm peak (Figures 1c and S1d,e). An increase in the UV absorbance of the RNAs, related to the well‐known hyperchromic effect upon denaturing and losing base‐pairings of nucleic acid chains, could also be observed through the temperature range (Figure 1d). Our earlier work with HOTAIR_140_ also confirmed that the RNA did not fold into G‐quadruplex conformations, as G4 peaks are missing from the NMR spectrum of HOTAIR_140_ (Szabó et al. 2022).

The G‐quadruplex content of the RNAs can also be assessed using ThT (Thioflavin T) fluorescence, as ThT shows a strongly elevated fluorescence when interacting with G‐quadruplex structures (Xu et al. 2016) with F/F_0_ ratios above 60 (de la Renaud Faverie et al. 2014). LiCl, which destabilizes G4s, is expected to achieve a significant reduction in the F/F_0_ ratio (Singh et al. 2025), if the elevated ThT signal originates from G4 structures. ThT fluorescence measured in KCl indicated that the shorter HOTAIR constructs (including HOTAIR_140_) are devoid of stable G‐quadruplexes, as their F/F_0_ ratios remained below 30 and LiCl did not induce a change in this value (Figure S2). The two longer HOTAIR RNAs did show an elevated F/F_0_ value, which was reduced approximately by 50% in the presence of LiCl. This could indicate the presence of G4 structures; however, it needs to be noted that the CD spectra of these RNAs are highly similar to the shorter constructs showing the presence of a typical A‐type double‐stranded helix structure (Figures S1c and S2), suggesting that the putative G4 content is probably not dominant. Also, while ThT is considered to be G4‐specific in RNAs, several studies indicate that cavities and mismatches in complex RNA structures can also result in elevated F/F_0_ ratios (Hanczyc et al. 2021; Lan et al. 2023). Therefore, based on our results, we concluded that while we cannot exclude the possibility of G quadruplexes existing in the larger RNA constructs, they are not dominating the structures, and the shorter RNAs fold into hairpins under the experimental conditions.

**FIGURE 2:**
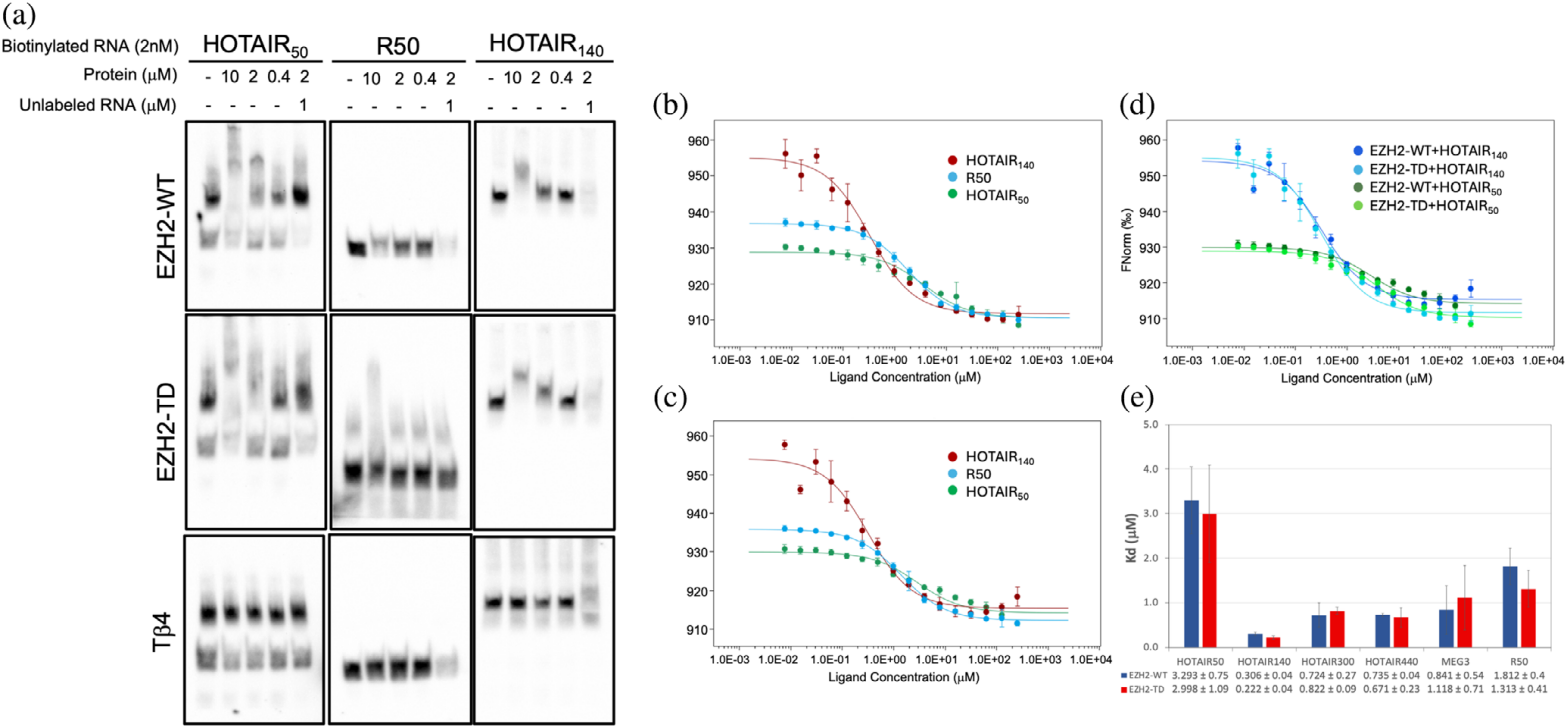
Binding of the WT and TD EZH2 loop to different RNAs. (a) Representative EMSA images with HOTAIR_50_, R50, and HOTAIR_140_. (b) MST binding curves of EZH2‐WT with HOTAIR_140_ (red), R50 (blue), and HOTAIR_50_ (green). (c) MST binding curves of EZH2‐TD with HOTAIR_140_ (red), R50 (blue), and HOTAIR_50_ (green). (d) Overlay of the binding curves of the WT and TD loops. Dark blue: EZH2‐WT + HOTAIR_140_, light blue: EZH2‐TD + HOTAIR_140_, dark green: EZH2‐WT + HOTAIR_50_, light green: EZH2‐TD + HOTAIR_50_. The average of at least five independent measurements is shown. (e) *K*_d_ values of EZH2‐WT (blue) and EZH2‐TD (red) with the different RNA constructs, calculated from the MST measurements.

To investigate how the phosphorylation of the T345 residue affects RNA recognition, we measured the interactions of the wild type (WT) and the phosphomimetic mutant (TD) version of the EZH2 loop with the different RNA constructs. Electrophoretic mobility shift assay (EMSA) confirmed that the WT and TD loop variants could interact with all tested HOTAIR constructs, in a similarly efficient manner (Figures 2a and S3a). The random sequence RNA (R50) showed a relatively small upward shift in the EMSA experiments, but the detectable shift and the intensity changes in the RNA bands indicate a clear binding pattern for the WT and the TD variants, compared to the non‐binding thymosin β4 (Tβ4) control (Figure 2a, middle column). MEG3 was also bound by the WT and the TD EZH2 loop, showing similar mobility shifts, indicating that the phosphorylation‐mimicking mutation did not affect the protein's RNA recognition specificity dramatically (Figure S3a). Tβ4, a disordered protein with no reported RNA binding capacity, could not interact with any of the tested RNAs (Figures 2a and S3a).

While EMSA experiments are useful in differentiating between binding and non‐binding events, they offer little information in terms of binding strength. In order to characterize the interactions in more detail, we performed microscale thermophoresis (MST), which provides quantitative data on the binding affinities. Results represented in Figure 2b–e show that the WT EZH2 loop bound all HOTAIR constructs with moderate affinities, ranging from 3.55 μM (HOTAIR_50_) to 0.3 μM (HOTAIR_140_). Interestingly, HOTAIR_140_ exhibits the highest affinity, while the longer constructs show somewhat reduced *K*_d_ values. As also indicated by the EMSA results, recognition is rather sequence‐independent, as R50 binds with slightly increased affinity compared to HOTAIR_50_, while MEG3 binds practically with the same *K*_d_ as HOTAIR_300_.

Confirming the results obtained with the EMSA experiments, the phosphomimetic mutation did not majorly affect the RNA binding measured with MST (Figures 2c and S3b), as no statistically significant differences could be detected between the *K*_d_ values of any RNAs (Figure S4). As for binding specificity, the TD mutation appears to somewhat reduce the specificity of the interactions, since the WT EZH2 loop presented more pronounced differences in affinity towards the different RNA constructs than the TD variant (Figure S4).

Interestingly, the binding mode of EZH2 loop (both the wild type and the TD variant) was slightly different when partnered with the specific HOTAIR_50_ versus the nonspecific R50 (Figure S5). As seen on the individual binding curves based on the T‐jump values (Figure S5a) (indicative of local changes), both of the EZH2 loop variants show an apparent kink in the curve and do not reach saturation even at the highest concentrations. When we consider the thermophoresis (Figure S5b), which provides information on the size, charge and solvation of the complexes, the two‐step behavior becomes clearer. However, the measured values were not consistent with a two‐site binding model, suggesting that instead of two binding events, there might be two structural states during the interaction. This appears to be a unique feature of the EZH2 loop–HOTAIR50 interaction, since no other HOTAIR construct showed similar behavior. Given that under physiological conditions EZH2 interacts with full‐length lncRNAs, there is little chance that this phenomenon has true biological relevance.

Since there was no significant difference between the binding affinities of the WT and the TD EZH2 loops, these results do not offer a mechanistic explanation of the observed alterations in the RNA binding of the phosphorylated protein (Kaneko et al. 2010). Therefore, we attempted to characterize the structural events that accompany the binding.

### 2.2 Structural changes of RNAs induced by binding to EZH2

The EZH2 loop studied here is highly disordered, as shown by our earlier NMR results (Szabó et al. 2022). Far‐UV CD measurements also show that the phosphomimetic mutation does not influence the structure of the protein (Figure S6), and it remains in the highly disordered state as it was verified on the CD spectra by the disordered–ordered classification tool (https://bestsel.elte.hu; Micsonai et al., 2022a). According to our earlier NMR measurements, RNA binding does not induce the large‐scale folding of either the WT or the TD loop (Szabó et al. 2022). As the NMR studies did not cover the structural changes of the RNAs, we aimed to study the behavior of the RNAs upon binding to the EZH2 loop by CD spectroscopy.

**FIGURE 3:**
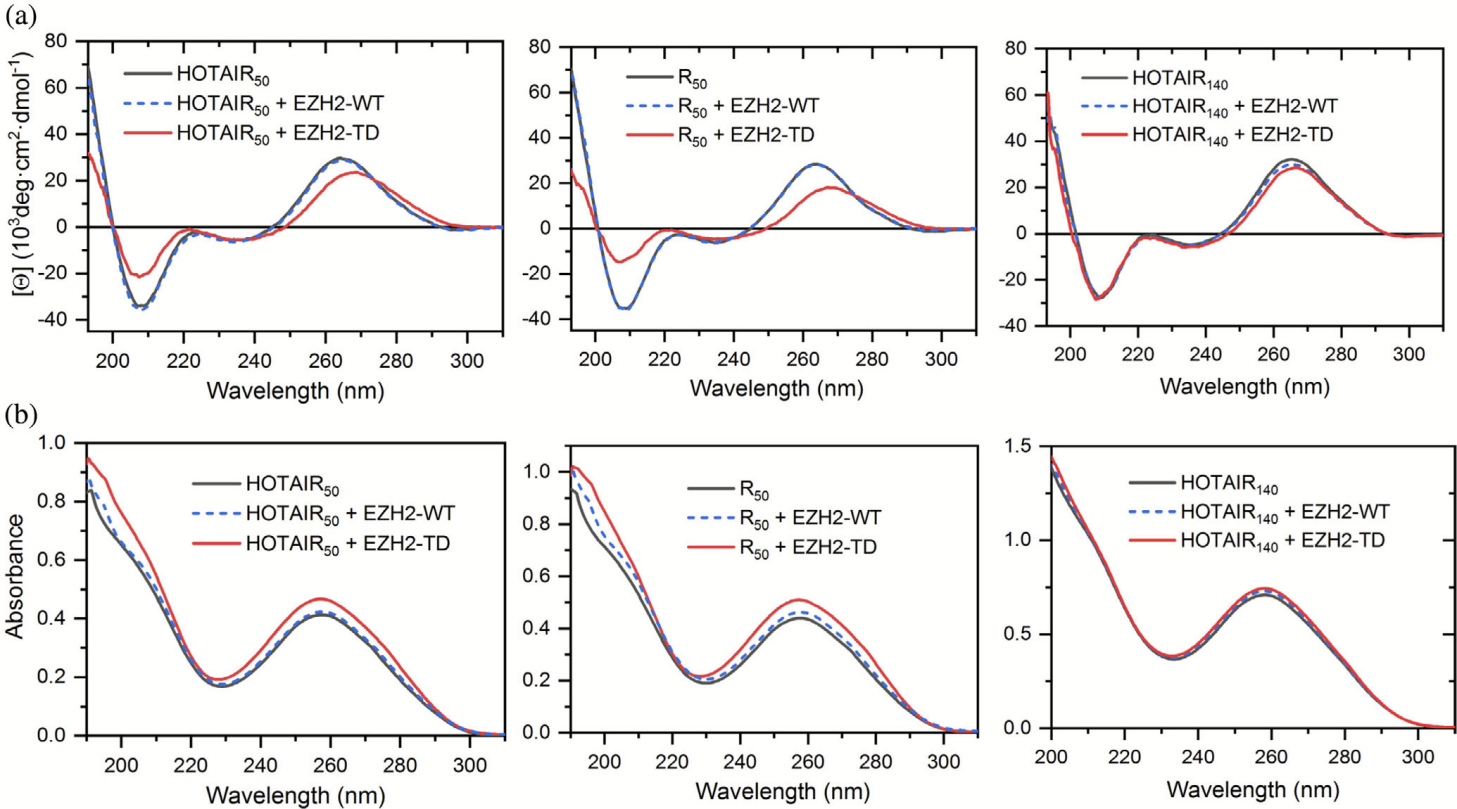
Spectral changes indicating transitions in the RNA structures upon binding to EZH2 loop variants. (a) CD spectra of the different HOTAIR constructs in isolation (black line) and in complex with the different EZH2 loop constructs: WT loop (blue dashed line) and TD loop (red line). (b) UV absorbance of the different HOTAIR constructs in isolation (black line) and in complex with the different EZH2 loop constructs: WT loop (blue dashed line) and TD loop (red line).

At room temperature, structural alterations in the RNAs could only be detected when they were bound by the TD mutant loop (Figure 3). While the binding of the WT loop did not result in major spectral changes, RNAs in complex with the phosphomimetic mutant exhibited a decrease and shift in the 265 nm CD peak, as well as a reduced amplitude in the 210 nm negative peak (Figure 3a). UV absorbance also indicated structural alterations in the RNAs when interacting with the TD loop, as evidenced by the increased absorbance at 260 nm (Figure 3b).

The two shorter RNAs, HOTAIR_50_ and R50, exhibited the most pronounced structural changes. In the case of the longer construct of 140 nucleotides (Figure 3), the effect was relatively smaller because of the noninteracting part of the larger RNA molecule.

Comparison of the CD and absorbance spectra to the ones recorded during thermal stability measurements (Figure 1c,d) reveals that these changes are caused by the partial unfolding of the RNAs, which mainly occurs when the phosphomimetic mutant loop is present as their molecular partner. Based on the increase in the UV absorbance, which is caused by the higher extinction coefficient of nucleotides of the single‐stranded RNA compared to the base‐paired ones (Nwokeoji et al. 2017), it was possible to estimate the number of disrupted base pairs upon HOTAIR‐EZH2 TD loop binding to be approximately 20 base pairs. This number is close to the predicted total number of base pairings in the shorter RNA constructs, explaining the stronger effect on their respective spectra. In the case of the longer constructs, the unfolding of 20 base pairs represents only a small portion of the total structural elements; therefore, the spectral effect is much less prominent (Figures 3 and S7).

**FIGURE 4:**
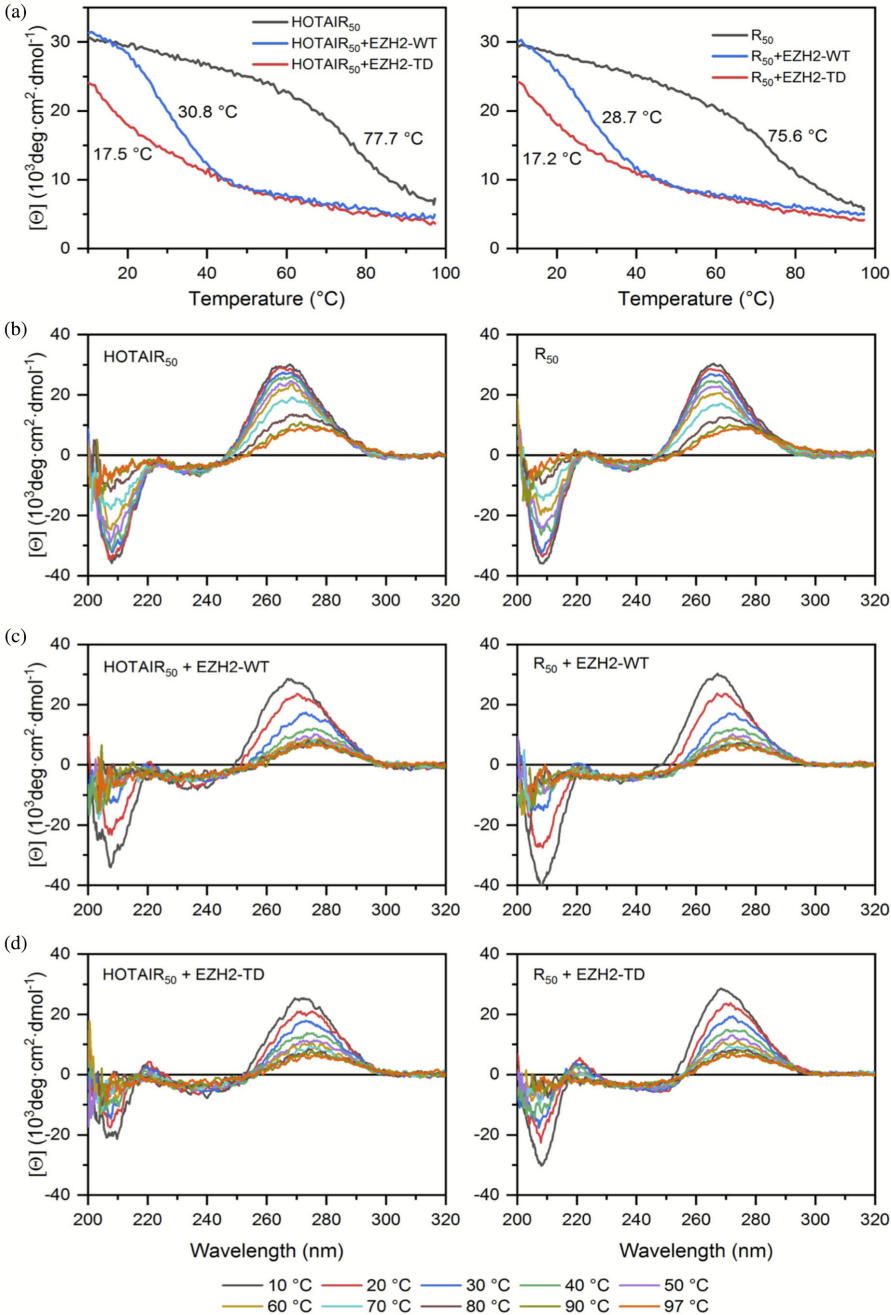
The effect of EZH2 loop variants on the stability of HOTAIR_50_ and R50 followed by thermal denaturation studies using CD spectroscopy. (a) Temperature scan of the free RNAs and in the presence of EZH2 loop variants was followed at 265 nm. Melting temperatures are indicated (calculated as described in section 4). (b–d) CD spectra were recorded at 10°C steps in the free forms (b), and in the presence of EZH2‐WT (c) and EZH2‐TD (d). Color coding is shown at the bottom.

To further dissect the spectral and structural effect of EZH2 variants on RNAs, we studied the thermal stability of HOTAIR_50_ and R50 in the presence of EZH2‐WT and TD loop, in comparison to the free RNAs. Upon heating the samples from 10 to 97°C, we followed the CD signal at 265 nm wavelength, where both RNAs show maximum (Figure 4a), and we also recorded the complete CD spectra with 10‐degree steps (Figure 4b–d). Without the protein partners, both RNAs exhibited high stability with melting temperatures above 70°C (Figure 4a). The addition of the EZH2 loop constructs however resulted in a dramatic decrease in stability for both of the RNAs. The melting temperatures for HOTAIR_50_ were reduced from 77.7°C to 30.8°C and 17.5°C in the case of the WT and TD loop, respectively. Similarly, melting temperatures of R50 were decreased from 75.6°C to 28.7°C (WT) and 17.2°C (TD). These results indicate that upon binding, both EZH2 loop variants destabilize the folded structure of RNA molecules, leading to disruption of the double helical structure. This effect is especially strong in the case of the phosphomimetic mutant, which unfolds the RNAs already at room temperature under the studied conditions.

## 3 DISCUSSION

The role of RNA binding in the regulation of the activity of PRC2 has long been puzzling for researchers. Different PRC2 complex components have been convincingly shown to interact with RNAs and RNA interactions are involved in the proper targeting of the complex (Dunn‐Davies et al. 2024). Yet, according to the currently accepted model, RNA binding and PRC2 methyltransferase activity are mutually exclusive, meaning that EZH2 needs to dissociate from RNAs in order to be enzymatically active (Beltran et al. 2016; Wang et al. 2017). The regulation and molecular mechanism of this switch are relatively unclear, as it is also known that the binding of PRC2 to lncRNAs such as HOTAIR activates the complex and overexpression of HOTAIR leads to elevated H3K27me3 levels in PRC2 target genes. A recent publication (Balas et al. 2021) described a compelling mechanism, where the low‐complexity region of hnRNP B1 induced the unfolding of a HOTAIR helix, facilitating the binding of HOTAIR to other RNAs. The resulting dsRNA presents much lower affinities towards EZH2; therefore, it allows for the activation of the PRC2 complex. While this model offers an explanation for the role of HOTAIR interactions in the regulation of PRC2 activity, it does not explain how the phosphorylation of EZH2 regulates its cell cycle‐dependent activation.

Our results clearly indicate that the disordered loop between A340 and A435 of EZH2 is capable of binding to different RNAs, in a rather sequence‐independent manner. While the literature seems to be in agreement that the loop region studied here does not participate in the interaction with G4 structures (Long et al. 2017; Song et al. 2023), the tested RNAs do not contain significant G4 structures and fold into mostly helical conformations. This indicates that this loop may be involved in the recognition of double‐stranded RNA regions in complex RNAs, probably in hairpin conformations, as suggested earlier (Kanhere et al. 2010; Li et al. 2021; Wu et al. 2013; Zhao et al. 2008). Contrary to our expectations however, the phosphomimetic mutation did not significantly influence the binding affinity, or the recognition specificity of the loop region. This may be somewhat surprising, given the marked effect on the RNA secondary structure of the phosphomimetic mutant. However, a similar *K*_d_ does not mean identical binding mechanisms. MST is an equilibrium technique measuring the equilibrium binding constants. Other techniques that determine the kinetic rate constants might point out the differences between the two EZH2 variants' RNA binding in association and dissociation rates. The similar affinity of the two variants might be a fairly possible coincidence. In the case of the TD variant, breaking base pairing requires an input of energy, which is unfavorable for the binding, and thus the overall affinity is decreased to the level of that of WT EZH2. We have to note that with increasing temperature, the effect of WT and TD EZH2 on the RNA structure converges to each other, and WT EZH2 is also able to disrupt base pairing (Figure 4).

Nevertheless, we could identify an important effect of the loop binding on the RNA structure. Apparently, binding of the disordered loop of EZH2 induces the unfolding of the double‐stranded RNAs, which is highly increased if the phosphomimetic mutation is present. This happens in a sequence‐independent manner, as the random sequence hairpin RNA (R50) showed a behavior similar to that of the HOTAIR_50_ RNA.

**FIGURE 5:**
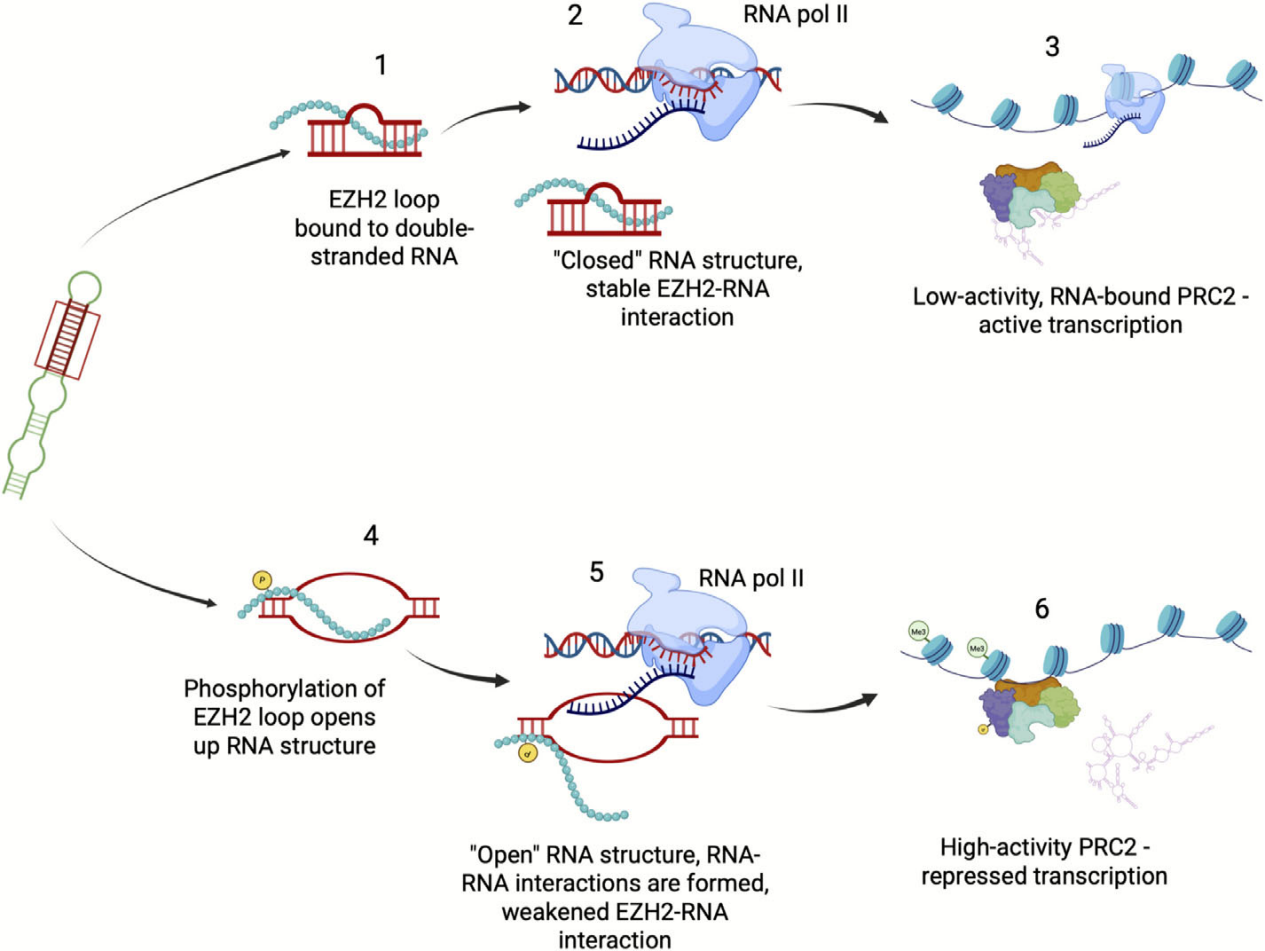
Schematic representation of the phosphorylation switch in the disordered loop of EZH2. The loop region is able to bind to dsRNA segments in HOTAIR and other complex RNAs. The unmodified loop has only a weak unfolding effect; therefore, it does not allow the full activation of the PRC2 complex (steps 1–3). Upon phosphorylation, the binding mechanism changes and induces the local unfolding of the RNAs, facilitating the interaction with nascent RNAs. The outcome of this type of binding is a release of PRC2 from the lncRNA and the full activation of the complex (steps 4–6). Created in BioRender. Tantos, A. (2025) https://BioRender.com/plag6d0

This observation leads us to propose the following model: the disordered loop of EZH2 may serve as a tether that keeps the PRC2 in close proximity to regulatory lncRNAs, such as HOTAIR, by interacting with hairpin structures. In this state, other RNA‐binding surfaces on EZH2 ensure stable RNA interaction and the PRC2 complex remains inactive (Figure 5, steps 1–3). When the loop gets phosphorylated in a cell cycle‐dependent manner, this phosphorylation switch changes its binding mode, increasing the unwinding of the double‐stranded RNA, enabling its base‐pairing with other RNA species. Similarly to the model proposed previously (Balas et al. 2021), this conformational change facilitates the dissociation of PRC2 from the RNA, and its subsequent activation (Figure 5, steps 4–6). It is important to note that our work concentrated on the isolated disordered loop of EZH2; therefore, our model cannot address additional regulatory mechanisms provided by the other RNA‐interacting regions of EZH2. These additional mechanisms can explain the overall weaker affinity to RNAs (Balas et al. 2021) of the whole PRC2 complex, while we did not detect a significant decrease in the affinity of the loop region.

Although a recent article raised questions regarding the physiological specificity of EZH2‐RNA interactions within cellular environments (Guo et al. 2024), several following articles argue for the physiological relevance of the RNA binding capacity of EZH2 (Cech et al. 2024; Deforzh et al. 2024; Lee et al. 2025; Lee and Lee 2024). Our findings support a specific RNA‐recognition mode for EZH2, as a specific post‐translational modification directly affects the binding mode of the protein. Our model also provides a plausible explanation for the cell‐cycle, CDK‐regulated activity changes of PRC2, without the need for the collaboration of other proteins, while not excluding the possibility of hnRNP B1‐mediated activation working in parallel.

### 3.1 Limitations of the study

Our work has three limitations that need to be taken into account when interpreting the results. The first limitation is that we used a phosphomimetic mutation instead of the phosphorylated version of the EZH2 loop, which may not fully mimic the effect of in vivo phosphorylation (Modic et al. 2024). While the T‐D mutation can reproduce the electrostatic effect of a phosphate group, it may fail to capture other chemical and structural features, like size, geometry, specific H‐bond formation and p*K*_a_. The combination of the structural and binding studies performed here and in our previous work can alleviate some of these discrepancies; experiments with a phosphorylated protein will provide a deeper understanding of the physiological consequences of the EZH2 loop phosphorylation. However, a predictive coarse‐grained model that was used to position‐specific effects of post‐translational modifications showed that phosphorylation may have a larger effect on protein–RNA interactions than the phosphomimetic mutation (Perdikari et al. 2021), suggesting that the phenomenon described here may be even stronger in the presence of a phosphorylated residue.

The second limitation is that our experiments were limited to the isolated disordered loop of EZH2, which excludes the assessment of the additional regulatory mechanisms provided by the other parts of the protein and/or PRC2 components.

The third limitation that needs to be accounted for is that only a hairpin control RNA was used and no unstructured RNA control was tested. This leaves open the possibility that the loop region of EZH2 may be able to recognize single‐stranded and unstructured RNA regions as well. Nevertheless, since the most pronounced difference in RNA binding between the unmodified and the phosphomimetic mutant loop was related to the unfolding of the double‐stranded RNA, this difference may be lost when interacting with other RNA structures.

## 4 METHODS

### 4.1 Protein cloning, expression, and purification

The same methods of protein overexpression and purification were used for both protein constructs, EZH2‐WT loop and EZH2 loop TD mutant. DNA sequence coding for WT protein (EZH2, UniProt: Q15910) was purchased from Eurofins Genomics (Ebersberg, Germany) and subcloned into pET22b(+) cloning vector. The TD mutant was generated using the site‐directed mutagenesis method (forward primer: ACGCATCAAAGACCCGCCT, reverse primer: TCAGCATCCATGGCCATC). The pET‐22b(+) vector containing the appropriate construct was transformed into competent *E. coli* BL21* (DE3) pLysS cells and grown in LB medium containing 0.05 mg/mL carbenicillin overnight at 37°C with shaking at 180 rpm. After inoculation with the starter cell culture into fresh LB medium containing 0.05 mg/mL carbenicillin, the cells were grown to OD_600_ = 0.8. Induction was done for 4 h at 30°C by 0.5 mM IPTG, then cells were pelleted by centrifugation (4000 rpm, 20 min, 4°C) and incubated at room temperature for 30 min in lysis buffer (20 mM Tris, 200 mM NaCl, 20 mM imidazole, 0.1% Triton X‐100, 1 mg/mL lysozyme, 50 U/mL nuclease (Pierce™ Universal Nuclease for Cell Lysis), pH 7.5, and EDTA‐free SIGMA*FAST* protease inhibitor cocktail tablets) with vigorous shaking. After sonication the cell debris was removed by centrifugation (12,100 rpm, 40 min, 4°C). The supernatant was filtered through a 0.2 μm nitrocellulose filter then purified over HisTrap HP column on an AKTA Explorer system using a gradient elution (Buffer A: 20 mM imidazole, 200 mM NaCl, 20 mM Tris, pH 7.5. Buffer B: 1M imidazole, 200 mM NaCl, 20 mM Tris, pH 7.5). Representative purification results are shown in Figure S8. Elution fractions containing sufficiently pure proteins were boiled for 10 min, and then pelleted by centrifugation (14,000 rpm, 20 min, 4°C). The supernatant was transferred to distilled water via buffer exchange on a HiPrep 26/10 desalting column (GE Healthcare, Chicago, IL). The elution fractions were lyophilized and stored at −20°C until use.

The negative control Thymosin beta 4 (Tβ4, UniProt: P62328) was produced as described earlier (Tantos et al. 2013).

### 4.2 RNA production and labelling

All HOTAIR (accession number NR_047517.1) DNA sequences cloned into a pEX‐A128 vector were purchased from Eurofins Genomics (Ebersberg, Germany). After 4 h digestion with EcoRV restriction enzyme at 37°C, the gel‐purified, linearized DNA template was used to synthesize RNA by in vitro transcription carried out with New England BioLabs HiScribe™ T7 Quick High Yield RNA Synthesis Kit (Ipswich, MA). After transcription, the remaining DNA template was eliminated with DNaseI treatment. RNA sample purification was carried out using Macherey‐Nagel NucleoSpin®RNA Clean‐up XS Kit (Duren, Germany). The quality and intactness of the purified transcription product were analyzed by native and formaldehyde agarose gel electrophoresis. Final RNA concentration was determined using Implen NanoPhotometer™ N60 Spectrophotometer (Munich, Germany). Purified RNA was stored at −80°C until usage in the presence of RNAINH‐RO Roche Protector RNase Inhibitor (20 U). Before use, the RNA sample was refolded by incubation at 75°C for 5 min and then allowed to cool to room temperature.

Maternally Expressed 3 (MEG3) lncRNA (NR_002766.2): pCI‐MEG3 was a gift from Anne Klibanski (Addgene plasmid #44727, Watertown, MA). Primers to obtain the DNA template by PCR reaction for in vitro transcription were as follows:

T7 RNA promoter region followed by the MEG3 forward primer: TAATACGACTCACTATAGGGGCAGAGAGGGAGCGCGCCTTGG.

MEG3 reverse primer: GATATCTTTTTGTTAAGACAGGAAACACATTTATTGAGAGC.

R50 was an artificial randomized RNA sequence. Fifty randomized sequences were generated using an in‐house PERL script. From the generated sequences the first was selected that does not occur naturally, which was tested using NCBI's Nucleotide BLAST (https://sky-blast.com/blast/n). No structural features were tested at the time of selection. Template DNA primers to obtain the RNA during the in vitro transcription were as follows:

T7 promoter region followed by 50 nt forward oligo: AAGAATGGCCTCGCGGAGGCATGCGTCATGCTAGCGTGCGGGGTACTCTT.

50 nt reverse oligo: AAGAGTACCCCGCACGCTAGCATGACGCATGCCTCCGCGAGGCCATTCTTCTATAGTGAGTCGTATTA.

Transcribed RNA: AAGAAUGGCCUCGCGGAGGCAUGCGUCAUGCUAGCGUGCGGGGUACUCUU.

All primers and oligonucleotides were purchased from Sigma‐Aldrich Ltd. (St. Louis, MO).

In the case of Cy5 labeled RNAs aminoallyl‐UTP‐Cy5 (Jena Bioscience, Thuringia, Germany) was added to the in vitro transcription.

Biotinylation of the RNAs was performed using Pierce™ RNA 3′ End Biotinylation Kit (Cat. No. 20160, Thermo Fisher Scientific, Waltham, UK) according to the instructions of the manufacturer. Overnight incubation at 16°C was applied for the ligation of the biotin label. Final RNA concentrations were determined using Implen NanoPhotometer™ N60 Spectrophotometer (Munich, Germany). Purified RNAs were stored at −80°C until use in the presence of RNAINH‐RO Roche Protector RNase Inhibitor (20 U).

### 4.3 RNA structure prediction

The secondary structure of the RNAs was predicted using the SSRTool web service (Yang et al. 2022) which uses a combination of different structure predictions (RNAprob, Fold, pKISS, RME_DMS, SHAPEKnots, MaxExpect, ProbKnot, MXSCARNA, RNAfold, RNALfold, pknots, Ipknot, RME_PARS). The significance level of functional interpretability was set to the default 0.05 value and the highest‐ranking variant was used for the purpose of this article.

The G‐quadruplex content was estimated by the QGRS Mapper online tool (Kikin et al. 2006), using the default settings.

### 4.4 Binding studies

#### 4.4.1 Electrophoretic mobility shift assay

LightShift® Chemiluminescent RNA EMSA Kit (Thermo Scientific, Cat. No. 20158, Thermo Fisher Scientific, Waltham, UK) was used for the EMSA experiments. Binding, electrophoresis, and detection of the tested RNAs with the proteins were carried out following the protocol of the kit. Briefly, proteins of varying concentrations were incubated with 1 or 2 nM of heat‐refolded RNAs for 30 min at room temperature, then loaded on 4 or 6% native polyacrylamide gels and run at 100 V in 0.5× TBE buffer for varying durations, depending on the size of the RNA. Then the RNA was transferred to nitrocellulose membranes using Trans‐Blot® Turbo™ Transfer System (Bio‐Rad, Hercules, CA) and crosslinked to the membrane by UV light. After proper washing and blocking, biotin‐labeled RNA was detected by chemiluminescence using Streptavidin‐Horseradish Peroxidase Conjugate.

#### 4.4.2 Microscale thermophoresis

RNA–protein binding assays were carried out on a Microscale Thermophoresis system (Monolith NT. 115 from NanoTemper Technologies, Munich, Germany). Standard treated capillaries (Cat. No. MO‐K002) were used for measurements. Instrument settings were as follows: Led power: 10–40%, MST power: 40%, before MST: 5 s, MST on: 30 s, after MST: 5 s, delay: 25 s.

Normalized fluorescence values at 1.25 s after turning on the IR laser were used as T‐jump and between 1.25 and 29.5 s as thermophoresis values.

RNA concentrations were set to give an initial raw fluorescence between 300 and 1000 counts and varied between 12.5 and 25 nM. All experiments were done at room temperature in a DEPC‐treated assay buffer (50 mM Tris pH 7.5, 150 mM KCl, 2.5 mM MgCl_2_, 1 mM DTT, 0.05% NP‐40).

### 4.5 CD spectroscopy

CD spectra of EZH2 variants, HOTAIR_50_, HOTAIR_140_ RNAs, and R50 of random sequence, were recorded on a conventional laboratory spectropolarimeter Jasco J‐810 (Jasco, Tokyo, Japan). The spectra were recorded in the wavelength range of 180–320 nm with a scanning speed of 20 nm/min, a bandwidth of 1 nm, an integration time of 4 s, and 6 scans accumulated. To study the structure of EZH2 variants, a protein concentration of ~1 mg/mL was used in a quartz cell with a 0.1 mm pathlength. CD spectra were quantitatively analyzed by the BeStSel method (Micsonai et al., 2022b; Micsonai et al. 2015; https://bestsel.elte.hu) and the tool for disordered–ordered classification (Micsonai et al., 2022a). The spectra of HOTAIR_50_, HOTAIR_140_, and R50 at a 5 μM concentration were measured in the absence or presence of equimolar EZH2‐WT or TD in a 1 mm quartz cell. Although at equimolar concentration ratios, the contribution of EZH2 to the CD spectra is negligible compared to the RNA spectra (especially in the 250–320 nm wavelength region), in the presence of proteins, the RNA spectra were produced by subtraction of the spectra of the EZH2 loop. CD spectra were recorded at 20°C or at 10°C steps in the 10–100°C temperature range. The temperature was controlled using a PTE Peltier unit. The thermal denaturation profile was fitted according to the Gibbs‐Helmholtz equation assuming a two‐state model, which is represented by a sigmoidal curve (Shih et al. 1995). HOTAIR_300_ and HOTAIR_440_ were measured by synchrotron radiation CD (SRCD) spectroscopy at the DISCO beamline of SOLEIL Synchrotron, France. SRCD spectra were recorded in CaF_2_ cells of 20–50 μm pathlengths using 20–200 μM RNA concentrations in different buffers at various temperatures.

### 4.6 ThT assay

Four micromolar stock solutions were prepared from all of the RNA constructs in DEPC‐treated KCl buffer (50 mM Tris pH 7.5, 150 mM KCl, 2.5 mM MgCl_2_, 1 mM DTT, 0.05% NP‐40) or LiCl buffer (50 mM Tris pH 7.5, 150 mM LiCl, 2.5 mM MgCl_2_, 1 mM DTT, 0.05% NP‐40) then refolded by incubation at 75°C for 5 min and allowed to cool to room temperature. Twenty microliter assay buffer, 10 μL RNA sample, and 10 μL 8 μM ThT stock solution in assay buffer were pipetted to the wells of a 96‐well black plate yielding a final concentration of 1 μM RNA and 2 μM ThT. The emission spectrum from 440 to 600 nm was recorded using a fluorescence spectrophotometer (BioTek Synergy Mx microplate reader) with an excitation wavelength of 425 nm. Both the excitation and emission bandwidths were set to 10 nm. The fluorescence intensity at 488 nm was used to determine the G‐quadruplex content of the RNA samples as a ratio of F (the intensity of the RNAs) and F_0_ (the intensity of ThT alone).

## AUTHOR CONTRIBUTIONS

**Beáta Szabó:** Conceptualization; investigation; data curation; formal analysis; visualization; writing – review and editing; resources; validation. **András Micsonai:** Methodology; investigation; visualization; writing – review and editing; funding acquisition; formal analysis. **József Kardos:** Conceptualization; investigation; funding acquisition; writing – review and editing; visualization; data curation; formal analysis. **Ágnes Tantos:** Conceptualization; funding acquisition; writing – original draft; methodology; visualization; writing – review and editing; supervision; resources; project administration.

## CONFLICT OF INTEREST STATEMENT

The authors declare no conflicts of interest.

## Supporting information


**Figure S1.** (a) Predicted G‐quadruplex structures (highlighted in yellow) in the different RNA sequences. Red frame: HOTAIR_50_, blue frame: HOTAIR_140_, green frame: HOTAIR_300_. The sequence within the blue and green frames represents HOTAIR_440_. R50 is shown separately. (b) Predicted secondary structures of HOTAIR_300_ and HOTAIR_440_. (c) Comparison of the CD spectra of the HOTAIR variants and R50, indicating similar structural features by the location of the spectral components. (d, e) Thermal unfolding of HOTAIR_300_ and HOTAIR_440_, respectively, followed by CD spectroscopy. Spectra were recorded from 10°C to 108°C at 7°C steps (from blue to red).
**Figure S2.** ThT fluorescence of the studied RNA constructs in KCl (dark gray) and in LiCl (light gray).
**Figure S3.** Binding of the WT and TD EZH2 loop to different RNA species. (a) Representative EMSA images with HOTAIR_440_, HOTAIR_300_, and MEG3. (b) MST binding curves of WT EZH2 loop with HOTAIR_300_ (red) HOTAIR_440_ (blue), and MEG3 (green). (c) Binding curves of WT EZH2 loop with HOTAIR_300_ (red), HOTAIR_440_ (blue), and MEG3 (green). The averages of at least three measurements are shown.
**Figure S4.** Statistical analysis of the MST results. Non‐paired Student *t*‐test was performed to determine the significant differences between the individual *K*_D_ values from the MST experiments. Non‐significant differences are marked with a “‐” sign. *p < 0.05; **p < 0.01; ***p < 0.005.
**Figure S5.** Differences in the binding of HOTAIR_50_ and R50 to EZH2 loop variants. (a) Binding curves based on the T‐jump values. (b) Binding curves calculated from thermophoresis.
**Figure S6.** CD spectra of the wild type EZH2 loop (black dashed line) and the TD mutant EZH2 loop (red line).
**Figure S7.** The spectra of the complexes indicate slight changes in protein structure and more pronounced changes in RNA structure. The red lines represent the theoretical CD spectra calculated from the numerical sum of the values of the individual molecules. Differences between the calculated and the measured spectra reveal structural changes upon binding. Since the proteins alone have CD signal close to 0 above 240 nm (see Figure S5), the signal above 240 nm informs exclusively about the RNA structure.
**Figure S8.** Coomassie blue stained SDS‐PAGE analysis of the purified recombinant His‐tagged proteins.
Lane 1, PageRuler™ Plus Prestained Protein Ladder (Thermo Scientific™, 26619); Lane 2, 10 μg WT EZH2 loop; Lane 3, 10 μg TD mutant EZH2 loop.

## Data Availability

The data that support the findings of this study are available from the corresponding author upon reasonable request.
