# Supplementary material for: Phosphorylation event changes the RNA binding mode of EZH2 disordered segment
Source: Protein Sci. 2025 Dec 23;35(1):e70391. doi: 10.1002/pro.70391 (PMC12723722; doi:10.1002/pro.70391)
Supplement: Supplementary file 1 — Figure S1. (a) Predicted G‐quadruplex structures (highlighted in yellow) in the different RNA sequences. Red frame: HOTAIR50, blue frame: HOTAIR140, green frame: HOTAIR300. The sequence within the blue and green frames represents HOTAIR440. R50 is shown separately. (b) Predicted secondary structures of HOTAIR300 and HOTAIR440. (c) Comparison of the CD spectra of the HOTAIR variants and R50, indicating similar structural features by the location of the spectral components. (d, e) Thermal unfolding of HOTAIR300 and HOTAIR440, respectively, followed by CD spectroscopy. Spectra were recorded from 10°C to 108°C at 7°C steps (from blue to red). Figure S2. ThT fluorescence of the studied RNA constructs in KCl (dark gray) and in LiCl (light gray). Figure S3. Binding of the WT and TD EZH2 loop to different RNA species. (a) Representative EMSA images with HOTAIR440, HOTAIR300, and MEG3. (b) MST binding curves of WT EZH2 loop with HOTAIR300 (red) HOTAIR440 (blue), and MEG3 (green). (c) Binding curves of WT EZH2 loop with HOTAIR300 (red), HOTAIR440 (blue), and MEG3 (green). The averages of at least three measurements are shown. Figure S4. Statistical analysis of the MST results. Non‐paired Student t‐test was performed to determine the significant differences between the individual K D values from the MST experiments. Non‐significant differences are marked with a “‐” sign. *p < 0.05; **p < 0.01; ***p < 0.005. Figure S5. Differences in the binding of HOTAIR50 and R50 to EZH2 loop variants. (a) Binding curves based on the T‐jump values. (b) Binding curves calculated from thermophoresis. Figure S6. CD spectra of the wild type EZH2 loop (black dashed line) and the TD mutant EZH2 loop (red line). Figure S7. The spectra of the complexes indicate slight changes in protein structure and more pronounced changes in RNA structure. The red lines represent the theoretical CD spectra calculated from the numerical sum of the values of the individual molecules. Differences between the calculate [file PRO-35-e70391-s001.pptx]

## Slide 1
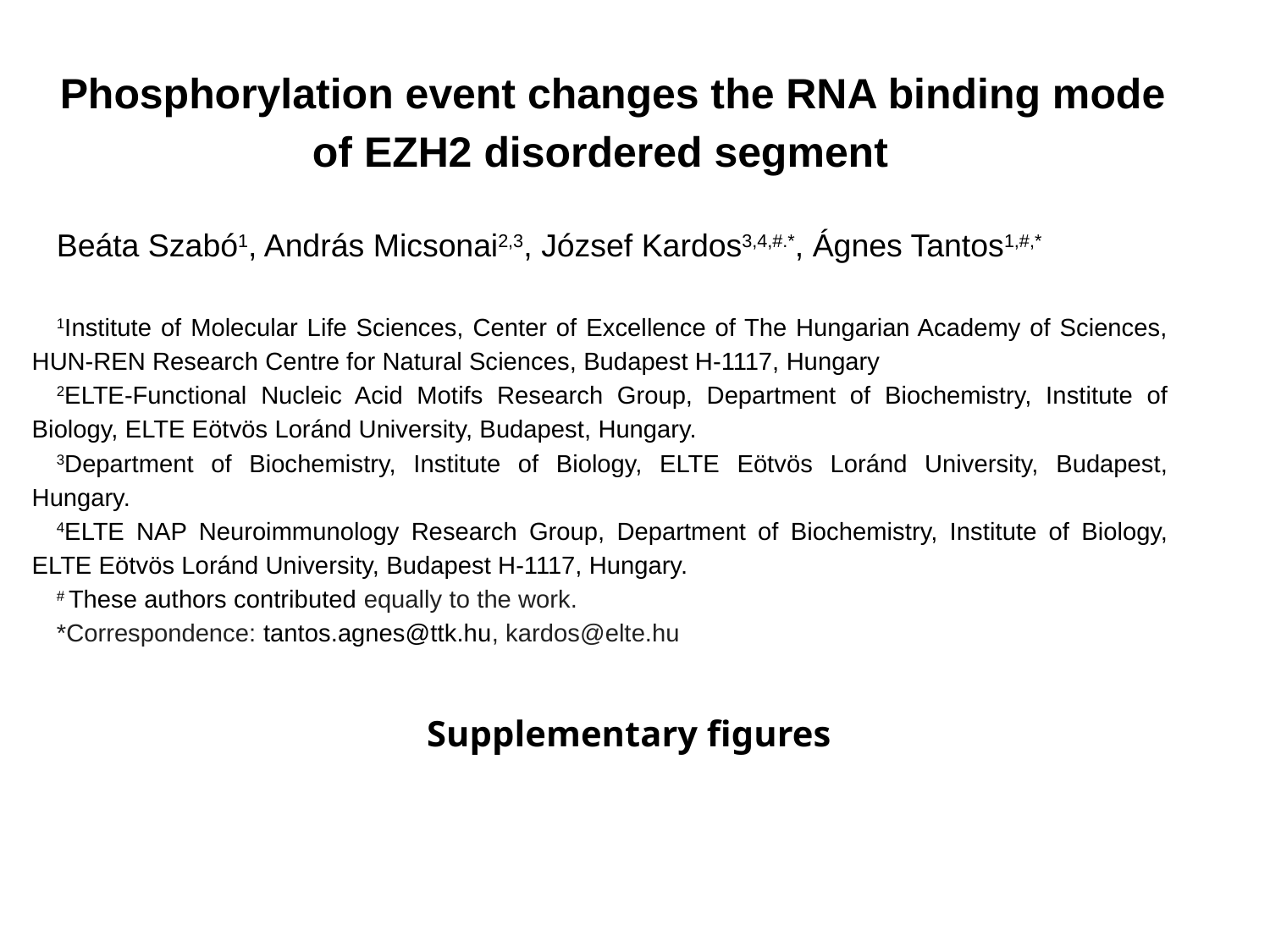

Phosphorylation event changes the RNA binding mode of EZH2 disordered segment
Beáta Szabó1, András Micsonai2,3, József Kardos3,4,#.*, Ágnes Tantos1,#,*
1Institute of Molecular Life Sciences, Center of Excellence of The Hungarian Academy of Sciences, HUN-REN Research Centre for Natural Sciences, Budapest H-1117, Hungary
2ELTE-Functional Nucleic Acid Motifs Research Group, Department of Biochemistry, Institute of Biology, ELTE Eötvös Loránd University, Budapest, Hungary.
3Department of Biochemistry, Institute of Biology, ELTE Eötvös Loránd University, Budapest, Hungary.
4ELTE NAP Neuroimmunology Research Group, Department of Biochemistry, Institute of Biology, ELTE Eötvös Loránd University, Budapest H-1117, Hungary.
# These authors contributed equally to the work.
*Correspondence: tantos.agnes@ttk.hu, kardos@elte.hu
Supplementary figures

## Slide 2
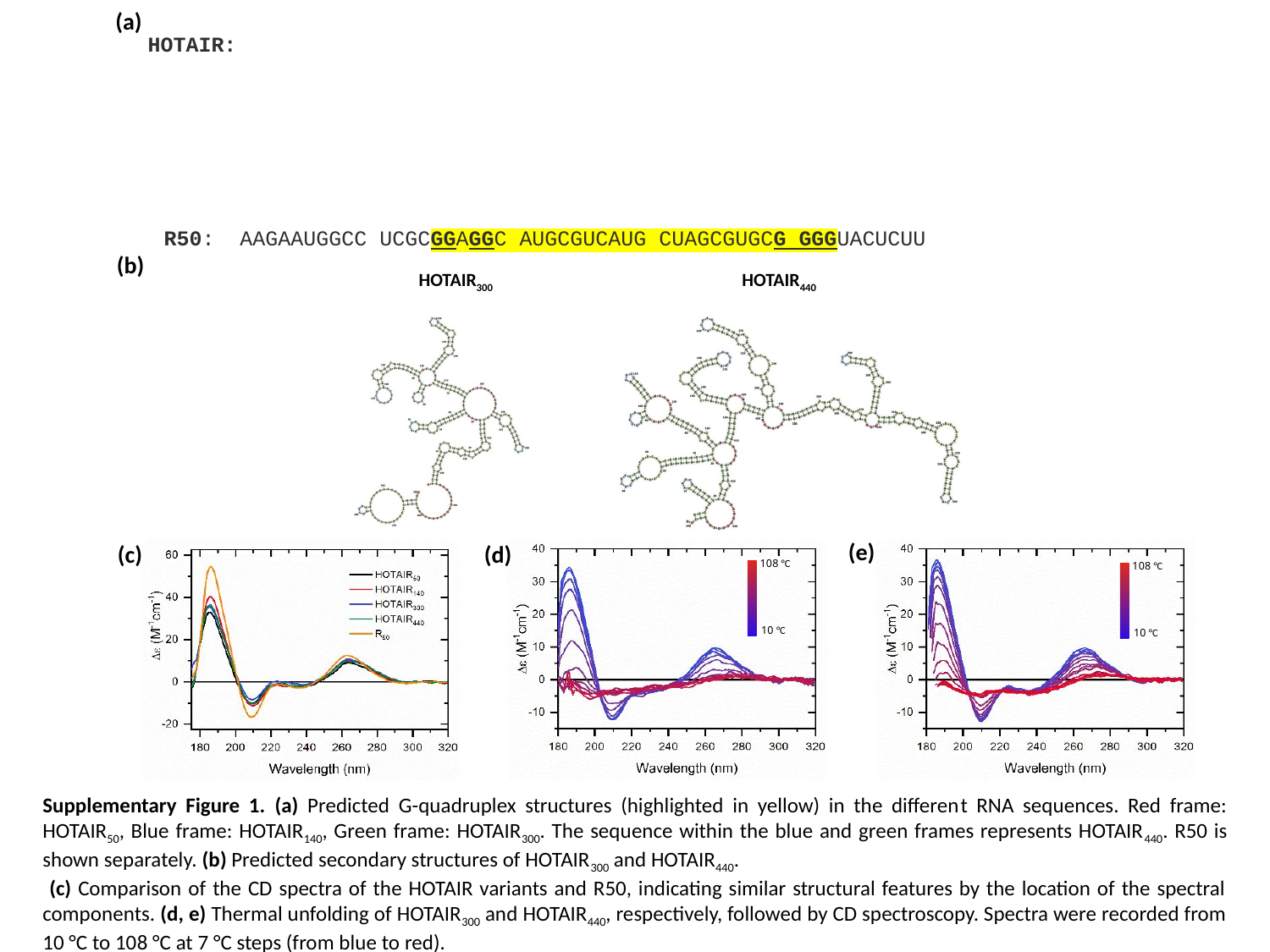

(a)
(b)
HOTAIR300
HOTAIR440
(e)
(c)
(d)
108 °C
10 °C
108 °C
10 °C
HOTAIR:
R50: AAGAAUGGCC UCGCGGAGGC AUGCGUCAUG CUAGCGUGCG GGGUACUCUU
Supplementary Figure 1. (a) Predicted G-quadruplex structures (highlighted in yellow) in the different RNA sequences. Red frame: HOTAIR50, Blue frame: HOTAIR140, Green frame: HOTAIR300. The sequence within the blue and green frames represents HOTAIR440. R50 is shown separately. (b) Predicted secondary structures of HOTAIR300 and HOTAIR440.
 (c) Comparison of the CD spectra of the HOTAIR variants and R50, indicating similar structural features by the location of the spectral components. (d, e) Thermal unfolding of HOTAIR300 and HOTAIR440, respectively, followed by CD spectroscopy. Spectra were recorded from 10 °C to 108 °C at 7 °C steps (from blue to red).

## Slide 3
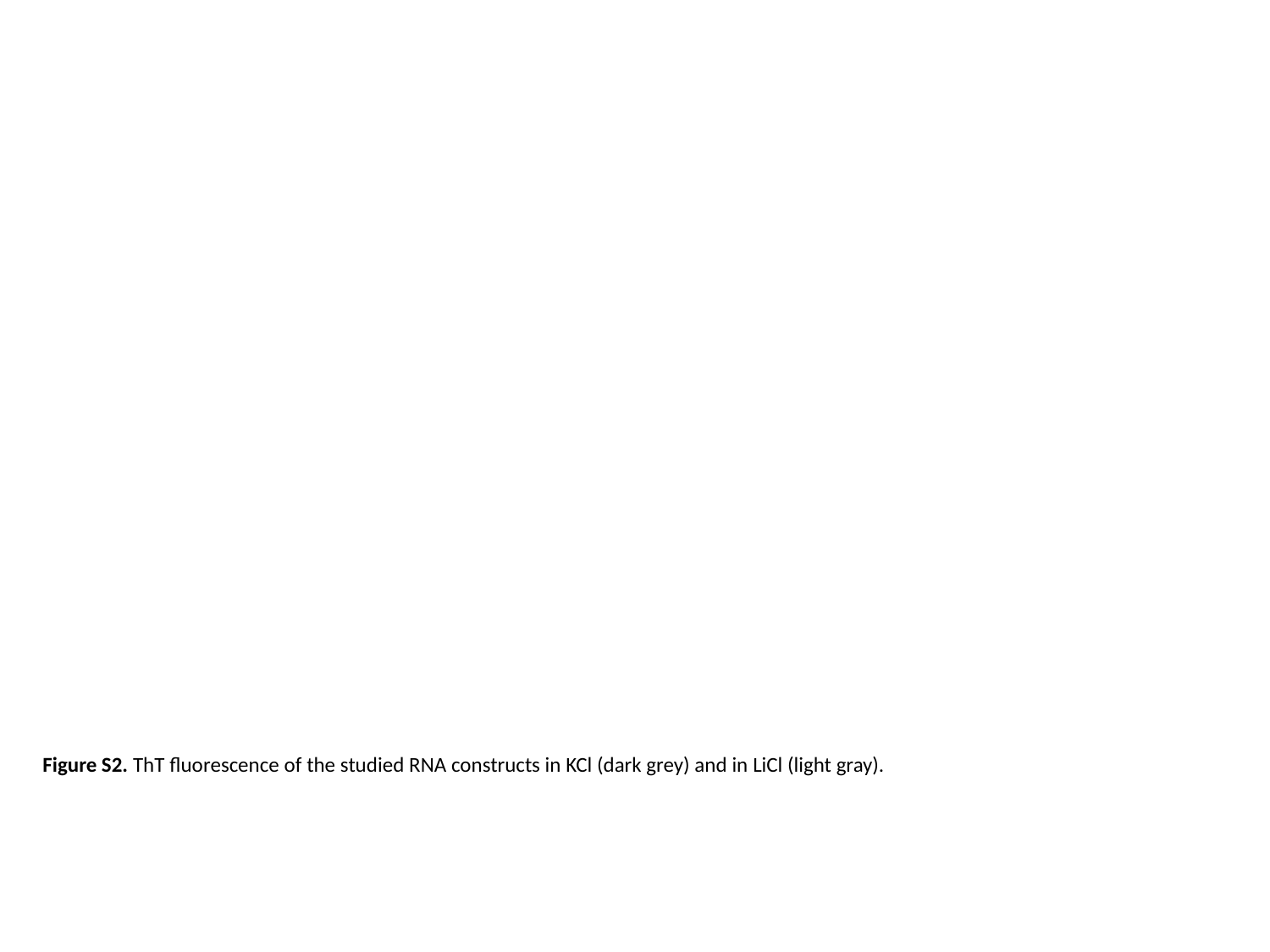

Figure S2. ThT fluorescence of the studied RNA constructs in KCl (dark grey) and in LiCl (light gray).

## Slide 4
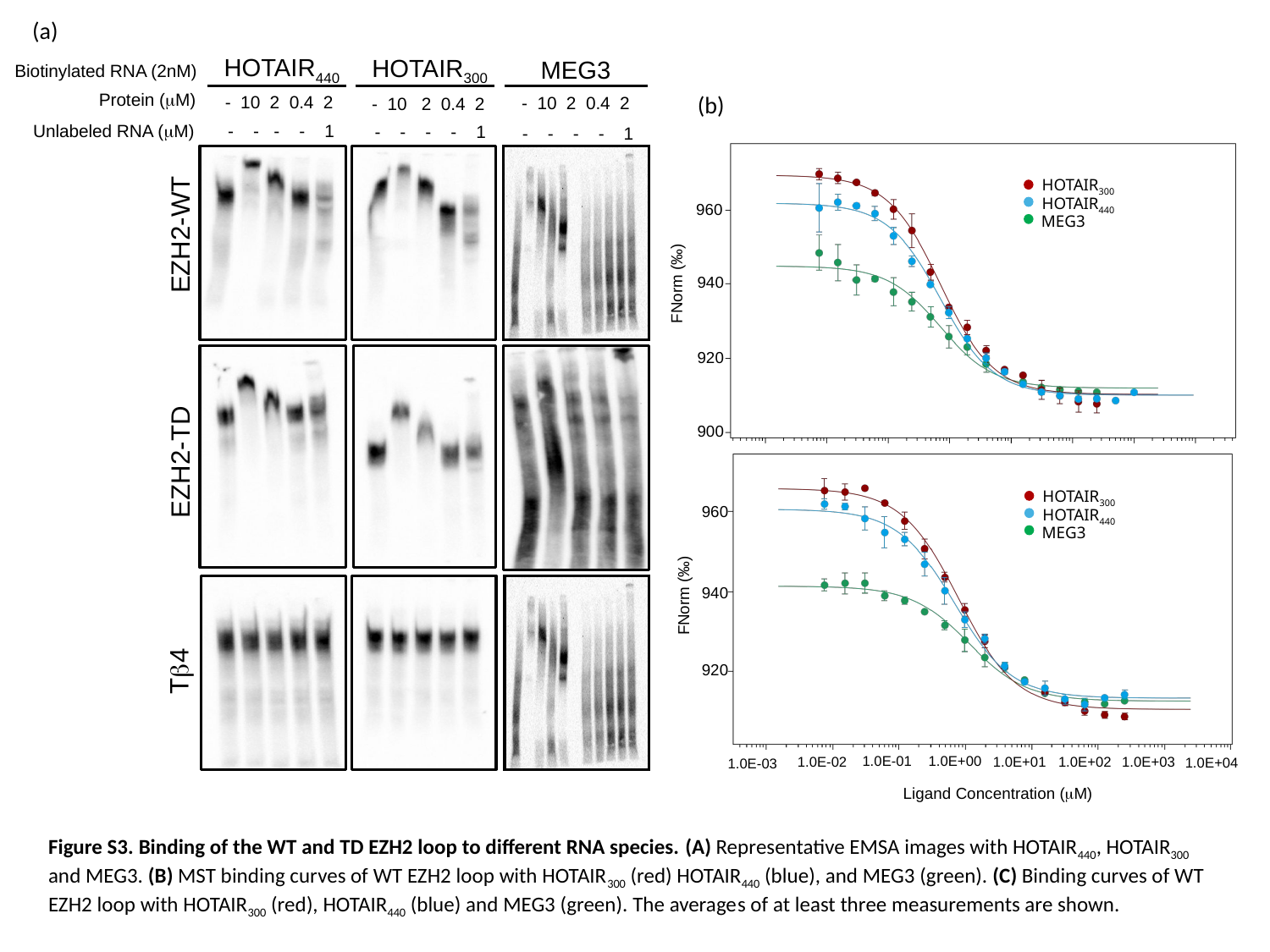

(a)
HOTAIR440
HOTAIR300
MEG3
Biotinylated RNA (2nM)
Protein (mM)
- 10 2 0.4 2
- - - - 1
Unlabeled RNA (mM)
- 10 2 0.4 2
 - 10 2 0.4 2
 - - - - 1
- - - - 1
EZH2-WT
EZH2-TD
960
940
920
FNorm (‰)
900
960
940
920
FNorm (‰)
Tb4
1.0E-01
1.0E+00
1.0E+02
1.0E-02
1.0E+01
1.0E+03
1.0E+04
1.0E-03
Ligand Concentration (mM)
(b)
HOTAIR300
HOTAIR440
MEG3
HOTAIR300
HOTAIR440
MEG3
Figure S3. Binding of the WT and TD EZH2 loop to different RNA species. (A) Representative EMSA images with HOTAIR440, HOTAIR300 and MEG3. (B) MST binding curves of WT EZH2 loop with HOTAIR300 (red) HOTAIR440 (blue), and MEG3 (green). (C) Binding curves of WT EZH2 loop with HOTAIR300 (red), HOTAIR440 (blue) and MEG3 (green). The averages of at least three measurements are shown.

## Slide 5
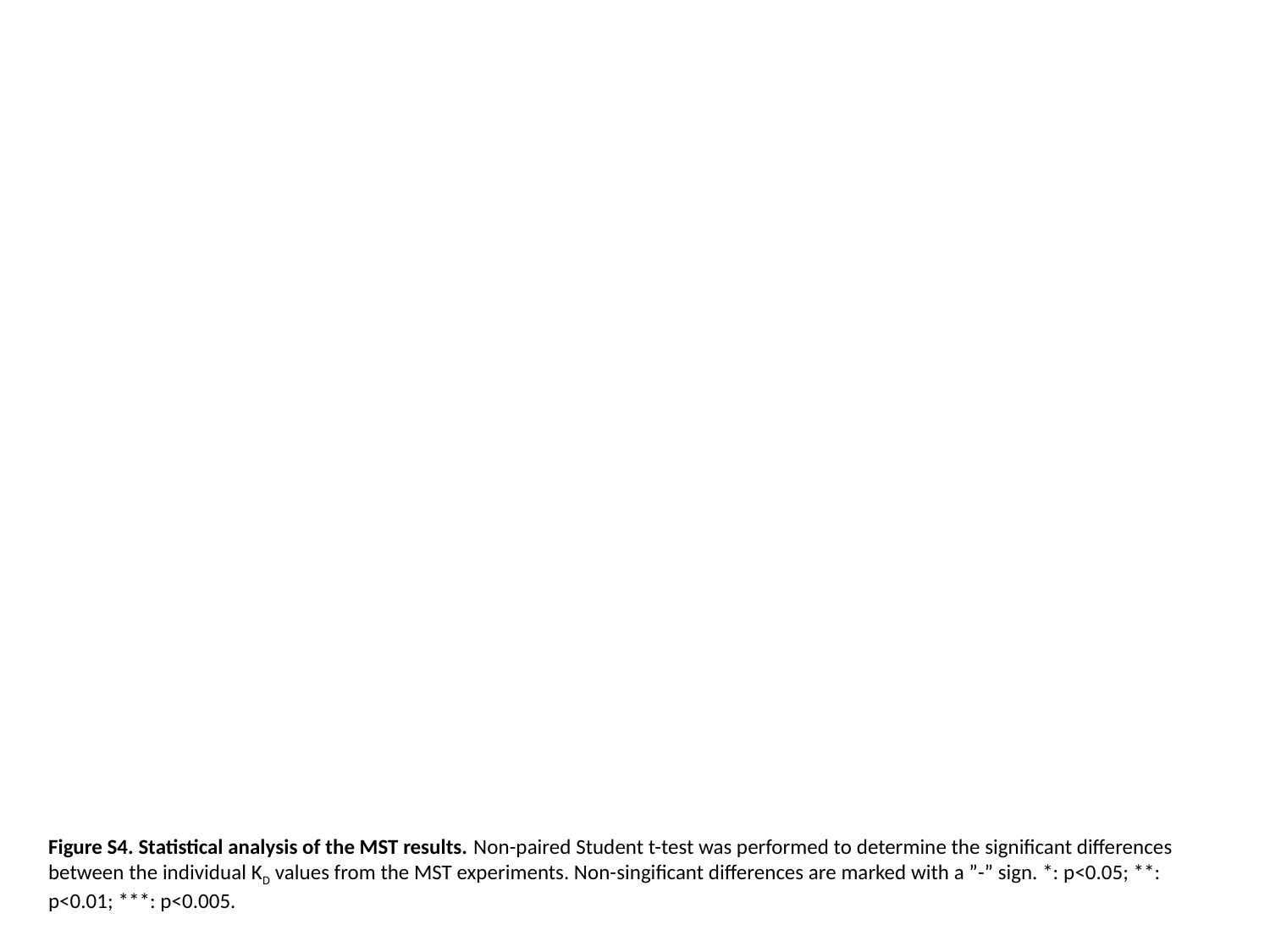

Figure S4. Statistical analysis of the MST results. Non-paired Student t-test was performed to determine the significant differences between the individual KD values from the MST experiments. Non-singificant differences are marked with a ”-” sign. *: p<0.05; **: p<0.01; ***: p<0.005.

## Slide 6
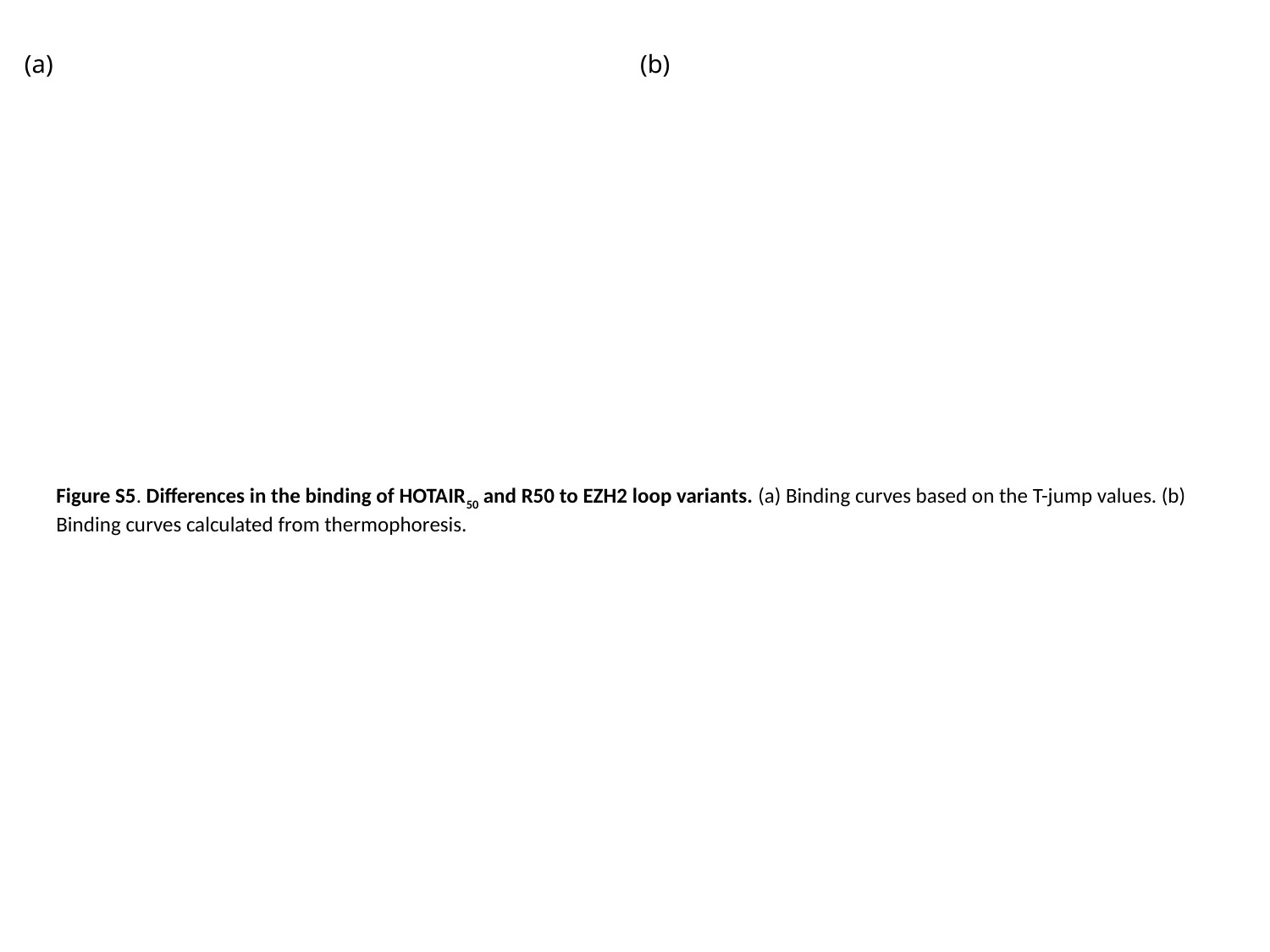

(a)
(b)
Figure S5. Differences in the binding of HOTAIR50 and R50 to EZH2 loop variants. (a) Binding curves based on the T-jump values. (b) Binding curves calculated from thermophoresis.

## Slide 7
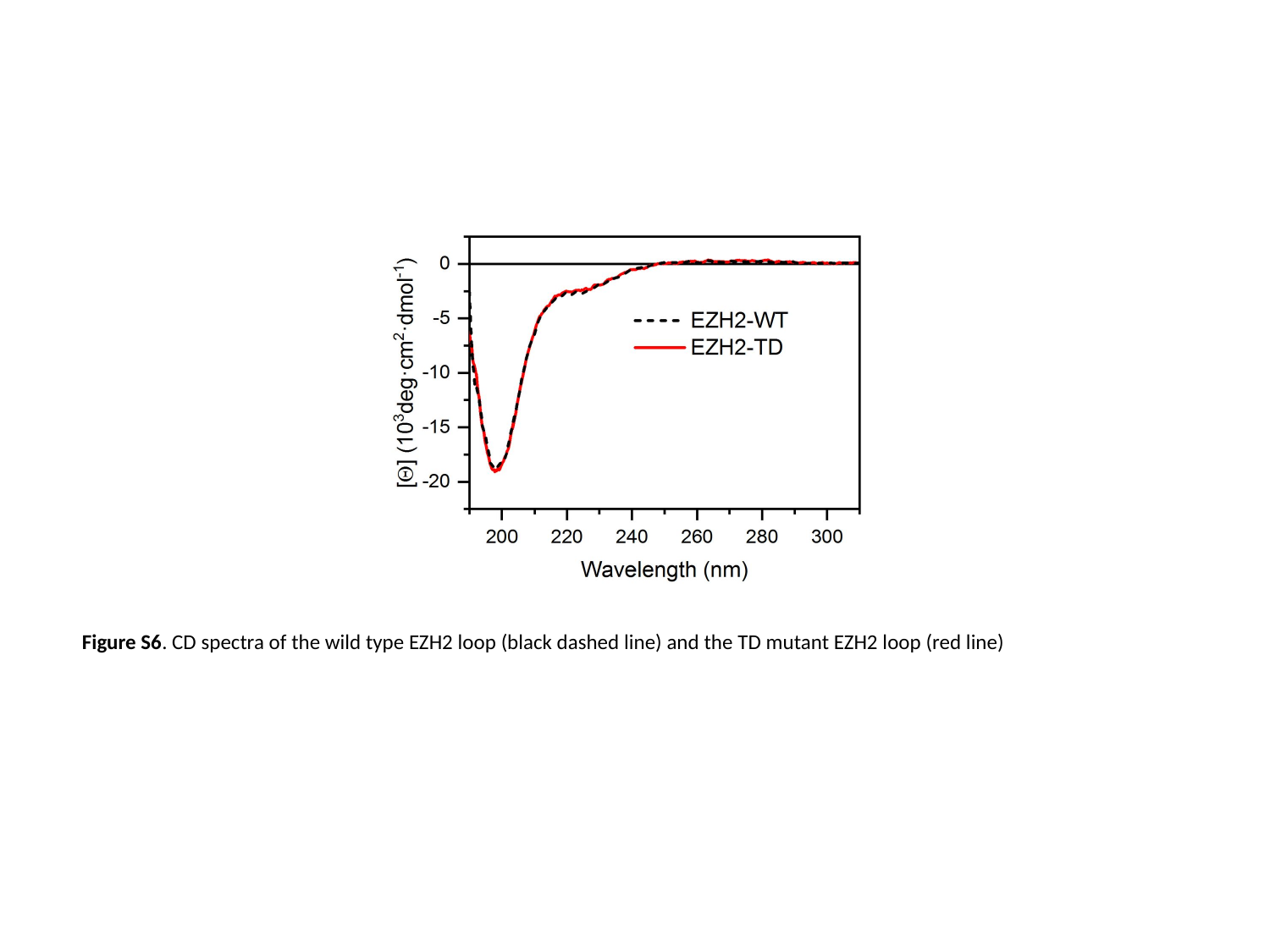

Figure S6. CD spectra of the wild type EZH2 loop (black dashed line) and the TD mutant EZH2 loop (red line)

## Slide 8
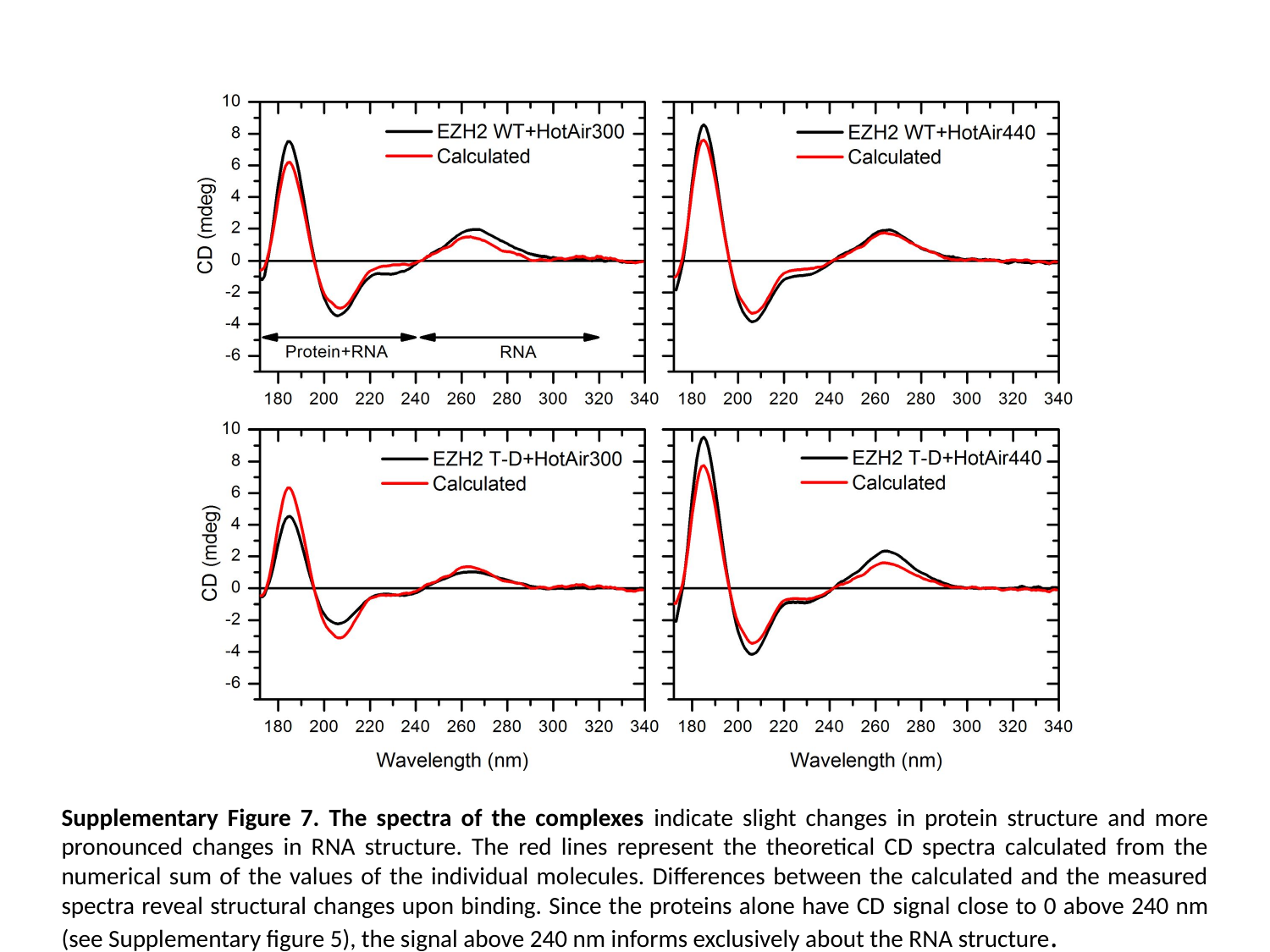

Supplementary Figure 7. The spectra of the complexes indicate slight changes in protein structure and more pronounced changes in RNA structure. The red lines represent the theoretical CD spectra calculated from the numerical sum of the values of the individual molecules. Differences between the calculated and the measured spectra reveal structural changes upon binding. Since the proteins alone have CD signal close to 0 above 240 nm (see Supplementary figure 5), the signal above 240 nm informs exclusively about the RNA structure.

## Slide 9
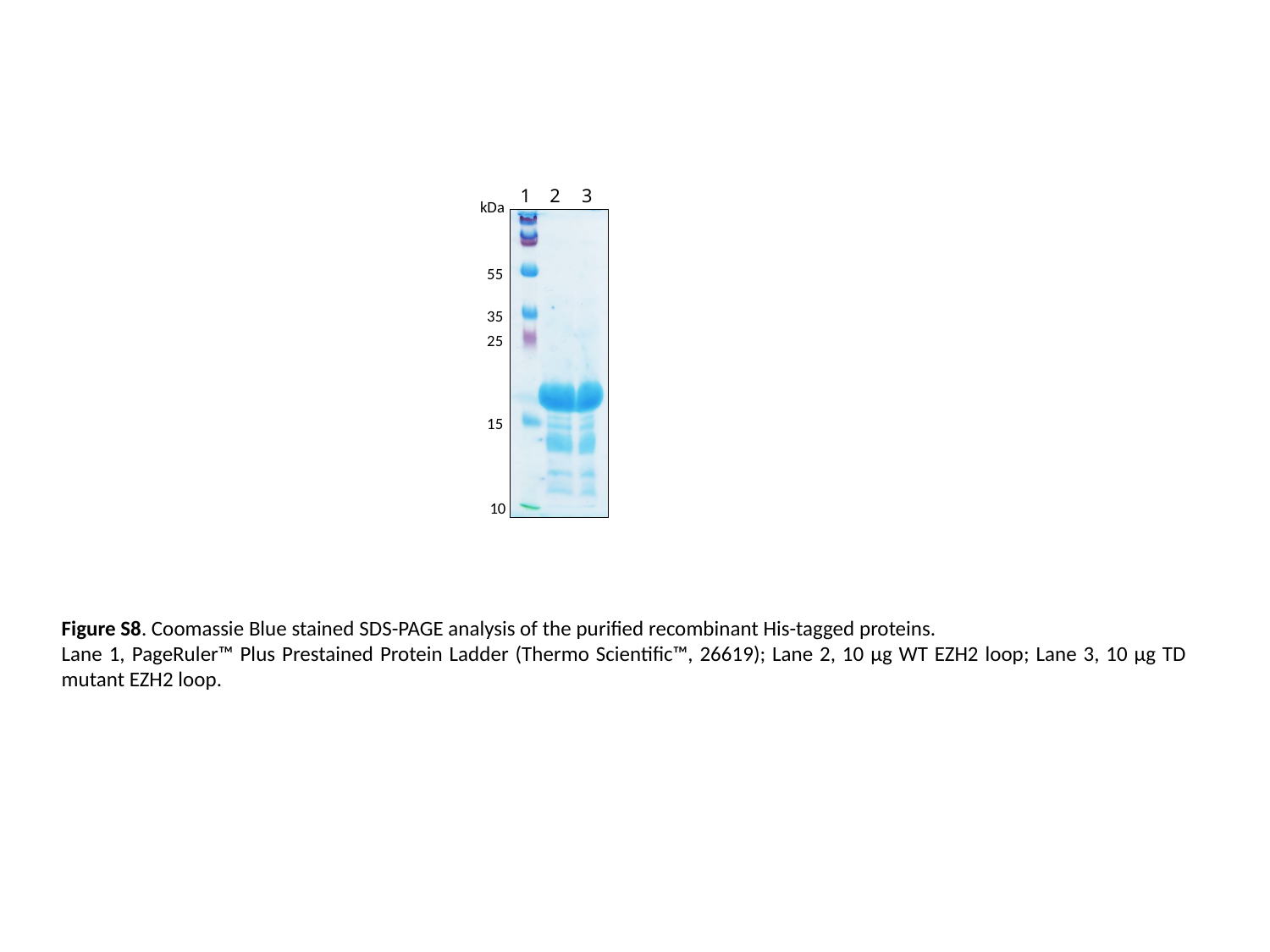

2
3
1
kDa
55
35
25
15
10
Figure S8. Coomassie Blue stained SDS-PAGE analysis of the purified recombinant His-tagged proteins.
Lane 1, PageRuler™ Plus Prestained Protein Ladder (Thermo Scientific™, 26619); Lane 2, 10 µg WT EZH2 loop; Lane 3, 10 µg TD mutant EZH2 loop.
